# The Role of AMPK Activation for Cardioprotection in Doxorubicin-Induced Cardiotoxicity

**DOI:** 10.1007/s10557-020-06941-x

**Published:** 2020-02-08

**Authors:** Kerstin N. Timm, Damian J. Tyler

**Affiliations:** 1 Department of Physiology Anatomy and Genetics, University of Oxford, Oxford, UK; 2 Oxford Centre for Clinical Magnetic Resonance Research, University of Oxford, Oxford, UK

**Keywords:** AMPK, Doxorubicin, Cardiotoxicity, AICAR, Metformin

## Abstract

Doxorubicin is a commonly used chemotherapeutic agent for the treatment of a range of cancers, but despite its success in improving cancer survival rates, doxorubicin is cardiotoxic and can lead to congestive heart failure. Therapeutic options for this patient group are limited to standard heart failure medications with the only drug specific for doxorubicin cardiotoxicity to reach FDA approval being dexrazoxane, an iron-chelating agent targeting oxidative stress. However, dexrazoxane has failed to live up to its expectations from preclinical studies while also bringing up concerns about its safety. Despite decades of research, the molecular mechanisms of doxorubicin cardiotoxicity are still poorly understood and oxidative stress is no longer considered to be the sole evil. Mitochondrial impairment, increased apoptosis, dysregulated autophagy and increased fibrosis have also been shown to be crucial players in doxorubicin cardiotoxicity. These cellular processes are all linked by one highly conserved intracellular kinase: adenosine monophosphate–activated protein kinase (AMPK). AMPK regulates mitochondrial biogenesis via PGC1α signalling, increases oxidative mitochondrial metabolism, decreases apoptosis through inhibition of mTOR signalling, increases autophagy through ULK1 and decreases fibrosis through inhibition of TGFβ signalling. AMPK therefore sits at the control point of many mechanisms shown to be involved in doxorubicin cardiotoxicity and cardiac AMPK signalling itself has been shown to be impaired by doxorubicin. In this review, we introduce different agents known to activate AMPK (metformin, statins, resveratrol, thiazolidinediones, AICAR, specific AMPK activators) as well as exercise and dietary restriction, and we discuss the existing evidence for their potential role in cardioprotection from doxorubicin cardiotoxicity.

## Introduction

### Doxorubicin-Induced Cardiotoxicity

UK cancer survival rates across all cancer types have doubled in adults and children over the last 40 years and now stand at 50% [1]. Some cancers such as breast cancer even show survival rates of 80% [2]. These improvements can, in part, be attributed to the impact of chemotherapeutics such as doxorubicin (DOX) [3], which is an anthracycline antibiotic first isolated from *Streptomyces peucetius* [4]. However, with increasing numbers of cancer survivors, long-term side effects of chemotherapeutics are becoming ever more apparent, and this is especially devastating for childhood cancer survivors [5]. Cardiotoxicity is one of the most severe side effects of chemotherapy and is defined as a reduction in left ventricular ejection fraction (LVEF) of greater than 10% to a value lower than 50% [6]. DOX in particular is severely cardiotoxic, causing congestive heart failure in ~ 5% of patients [7], though the incidence of DOX cardiotoxicity is dose dependent and can range from 3 to 18% [8]. This nowadays limits the recommended maximum lifetime dose of DOX to < 450 mg/m^2^ to lessen the risk of cardiotoxic side effects [9]. DOX also shows sex-related difference in cardiotoxicity in both patients and in preclinical models [10], with female cancer patients before puberty and after menopause most susceptible to DOX-induced cardiotoxicity [11]. Details on incidence, risk factors, timing and outcomes in cancer patients treated with DOX are reviewed elsewhere [12].

The prognosis in patients with DOX-induced congestive heart failure is poor [13]. Therefore, patients on DOX chemotherapy are monitored regularly to assess cardiac LVEF and chemotherapy cessation is recommended when values drop below 40% [14]. PEGylated liposomal formulations of DOX can reduce the incidence of cardiotoxicity, though they have been associated with other side effects such as skin toxicity [15]. Currently, there are no cardiotoxicity-specific treatments, neither prophylactic nor curative, and cardioprotective drugs trialled in patients to treat DOX cardiotoxicity are sparse and include standard heart failure medications such as renin angiotensin system blockers and beta blockers [12, 16]. Therefore, there is an unmet clinical need for more targeted cardioprotective therapy for cancer survivors with DOX cardiotoxicity, or, even more importantly, prophylactic treatment for cancer patients receiving DOX to minimise the incidence of cardiotoxic side effects leading to heart failure. In order to hit a specific target, detailed knowledge of the underlying molecular mechanisms of DOX cardiotoxicity is required.

### Molecular Mechanisms of DOX-Induced Cardiotoxicity

**Fig. 1:**
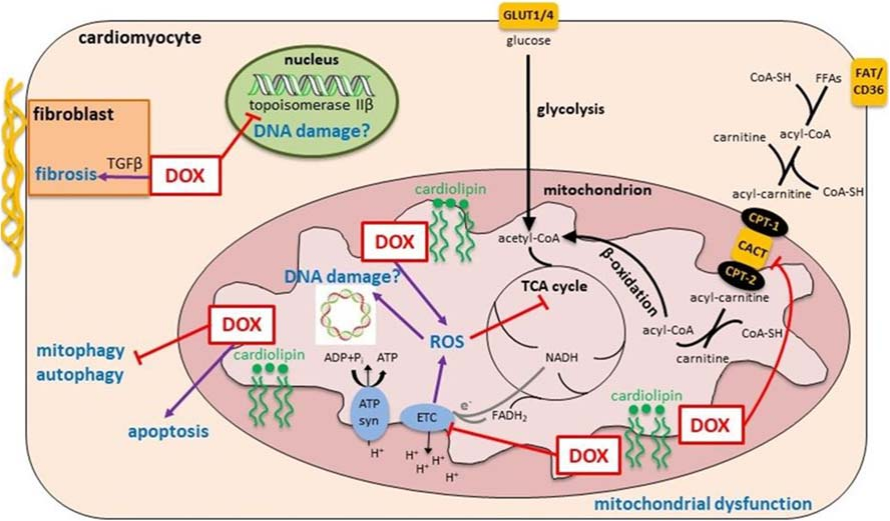
Molecular mechanisms of doxorubicin-induced cardiotoxicity. Doxorubicin (DOX) preferentially binds to cardiolipin in the inner mitochondrial membrane. Through its proximity to mitochondrial membrane proteins, DOX interferes with the electron transport chain (ETC), which is thought to contribute to reactive oxygen species (ROS) generation and mitochondrial dysfunction. DOX also inhibits uptake of free fatty acids (FFAs) into mitochondria by inhibiting the carnitine acyl-carnitine translocase. ROS can furthermore be directly produced by DOX through redox cycling on the quinone moiety and by Fenton reaction with molecular iron. ROS inhibits several enzymes in the tricarboxylic acid (TCA) cycle and may damage mitochondrial DNA. DOX furthermore inhibits mitophagy and autophagy and induces apoptosis. In the nucleus, DOX inhibits topoisomerase IIβ, which may lead to DNA damage. In fibroblasts (inset), DOX triggers TGFβ signalling, which induces fibrosis. ATP syn, ATP synthase; CoA-SH, coenzyme A; CPT-1/2, carnitine palmitoyltransferase; FAT, fatty acid transporter; GLUT, glucose transporter

DOX accumulates in the heart by binding to cardiolipin in the inner mitochondrial membrane [17]. DOX clearance from the myocardium lags far behind plasma clearance [18], which may explain why the heart is so susceptible to DOX. Different mechanisms have been proposed for the cardiotoxic effect of DOX [19] (Fig. 1). The most popular and widely researched mechanism of DOX cardiotoxicity is oxidative stress, which has been reviewed in detail elsewhere [20, 21]. In brief, reactive oxygen species are thought to be generated by different mechanisms, including Fenton reaction with molecular iron and redox cycling on the quinone moiety of DOX. However, it has already been suggested that oxidative stress may not be at the core of DOX-induced cardiotoxicity as iron-chelating agents, such as dexrazoxane, that should reduce oxidative stress have been only partially efficacious in patients or have even proven unsuccessful [22]. In addition, dexrazoxane is associated with some safety concerns, at least in paediatric patients [23], making the hunt for other specific cardioprotective agents all the more important.

The anti-cancer action of DOX is thought to be mainly due to inhibition of topoisomerase IIα, which leads to DNA double strand breaks and initiation of apoptosis. Topoisomerase IIα does not exist in cardiomyocytes; however, a role for topoisomerase IIβ in DOX cardiotoxicity has recently been proposed, potentially leading to DNA damage and mitochondrial impairment [24]. Mitochondria make up around 50% of the cardiomyocyte volume and are vitally important for energy generation through the sequential processes of tricarboxylic acid (TCA) cycle, electron transport chain and oxidative phosphorylation. Long-chain fatty acids and glucose are the main respiratory fuels for ATP generation in the heart. As DOX binds to cardiolipin, this perturbs protein function in the inner mitochondrial membrane, and, therefore, energy generation. Mitochondrial dysfunction in DOX cardiotoxicity has been reviewed comprehensively elsewhere [25]. Further recent review articles cover the role of autophagy [26, 27] and mitophagy [28] in DOX cardiotoxicity. Moreover, fibrosis plays a vital part in structural remodelling in DOX cardiotoxicity, initiated through TGFβ signalling, and this has been comprehensively reviewed recently [29]. It is evident that DOX cardiotoxicity is not attributable to one single target, rather, a multitude of proteins and pathways is affected and modulated by DOX. Therefore, it is unlikely that one specific drug targeting any specific protein alone will show meaningful clinical benefit in DOX cardiotoxicity. However, some of the above-described mechanisms are linked by a cellular master regulator, the adenosine monophosphate–activated protein kinase (AMPK), which regulates mitochondrial biogenesis and function as well as autophagy and fibrosis [30]. AMPK therefore provides a link between at least some of the proposed molecular mechanisms of DOX cardiotoxicity outlined above. In this review, we will introduce AMPK and its possible role in DOX cardiotoxicity and we will describe how AMPK activation by different means has already shown cardioprotection in animal models and in patients receiving DOX-based chemotherapy.

## AMPK

### Structure and Function of AMPK

**Fig. 2:**
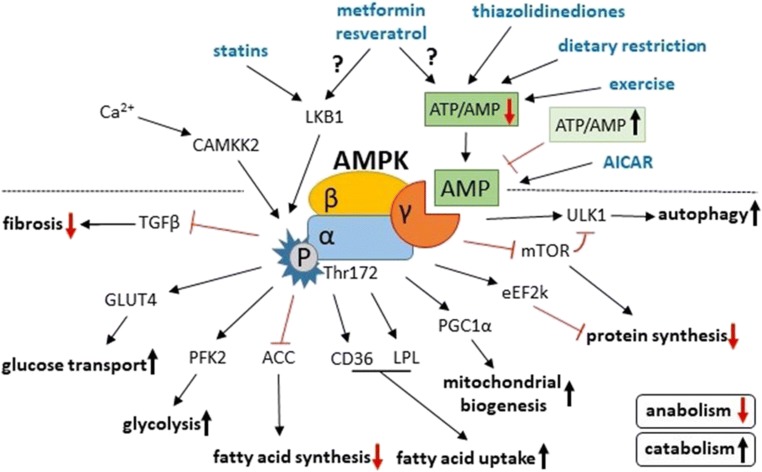
AMPK activation and downstream effects in the heart. AMPK is activated by phosphorylation on its α subunit (Thr172) via calcium/calmodulin-dependent protein kinase kinase II (CAMKK2) and liver kinase B1 (LKB1). AMP acts as an allosteric regulator on the γ subunit, preventing dephosphorylation of AMPK. This occurs when the ATP to AMP ratio is low, signalling a low-energy state. A low ATP to AMP ratio is also present during exercise and dietary restriction and can be pharmacologically achieved with thiazolidinediones and potentially with metformin and resveratrol. 5-aminoimidazole-4-carboxamide riboside (AICAR) is an AMPK mimetic and thus does not rely on the ATP to AMP ratio for AMPK activation. LKB1 can be activated through sirtuin 1-mediated deacetylation by statins and potentially with metformin and resveratrol. Downstream targets of phosphorylated AMPK overall promote catabolic processes such as glucose uptake and glycolysis, fatty acid uptake, mitochondrial biogenesis and autophagy and inhibit anabolic processes such as protein synthesis (and fibrosis). ACC, acetyl-CoA carboxylase; CD36, fatty acid transporter; eEF2k, eukaryotic elongation factor 2 kinase; GLUT4, glucose transporter 4; LPL, lipoprotein lipase; mTOR, mammalian target of rapamycin; PFK2, phosphofructokinase 2; PGC1α, peroxisome proliferator–activated receptor-gamma coactivator 1α; TGFβ, transforming growth factor β; ULK1, Unc-51 like autophagy activating kinase

AMPK is conserved across the animal kingdom, exists as orthologues in yeast and plants, and consists of different isoforms with tissue-specific expression [31]. AMPK is a heterotrimeric protein complex with a catalytic α and regulatory β and γ domains (Fig. 2). All three subunits display 2 different isoforms in the heart [32]. The α1 subunit is mostly expressed in cardiac endothelial cells [33] and the α2 subunit in cardiomyocytes [34]. AMPK is activated upon phosphorylation of its α subunit at Thr172 by one of two upstream kinases: liver kinase B1 (LKB1) [35] and calcium/calmodulin-dependent protein kinase kinase II (CAMKK2) [36]. Phosphorylation of the Thr172 residue on the catalytic α1 subunit is thought to be the predominant mechanism of AMPK activation; nonetheless, several other phosphorylation sites have been shown to exist on AMPK (Thr258, Ser485 on α1, Ser491 on α2 and Ser96, Ser101 and Ser108 on β1) [37]. Phosphorylation of the α1 and α2 subunits leads to similar activity, though the downstream substrates differ [38]. This means that activation of AMPK by differential phosphorylation on distinct threonine and serine residues may modulate AMPK activity more subtly than a simple binary ‘on-and-off’, as well as allowing subcellular localisation or differential substrate recognition of AMPK. For instance, the phosphorylated AMPKα2 containing the β2 isoform can translocate into the nucleus, activating gene expression, for example the peroxisome proliferator-activated receptor α (PPARα) [39]. AMP binding to the regulatory γ subunit shields AMPK from dephosphorylation [40] by its three phosphatases: protein phosphatase 2A (PP2A) [41], protein phosphatase 2C (PP2C) [42] and Mg^2+^/Mn^2+^-dependent protein phosphatase 1E (PPM1E) [43].

Activated AMPK phosphorylates a multitude of downstream effectors, including those involved in metabolic processes, apoptosis and autophagy [44]. AMPK is a master regulator of cellular energy homeostasis and mitochondrial health [45]. In the heart, this is largely due to its activation of both glucose and fatty acid uptake and oxidation [46, 47] when cellular AMP levels rise, signalling a low-energy state. AMPK activation also leads to increased mitochondrial biogenesis through increased expression of PGC1α [48]. The cardiac targets of AMPK are summarised in [32] and include metabolic enzymes such as phosphofructokinase-2, metabolite transporters such as the fatty acid transporter CD36 and signalling molecules such as mTOR, which is involved in the regulation of metabolism, protein synthesis and autophagy in the heart [49]. AMPK activation furthermore reduces hepatic lipogenesis and increases hepatic β-oxidation and reduces adipocyte lipolysis and lipogenesis, which has made it an attractive drug target for the treatment of type 2 diabetes [50]. Despite its obvious regulation of lipid metabolism in the liver and adipose tissue, AMPK activation has been specifically associated with the beneficial effects of some anti-diabetic drugs on cardiovascular diseases [51]. In addition, AMPK activation has been proposed as a therapeutic target in heart failure [52], where its cardioprotective role is thought to be due to mechanisms beyond regulation of cellular energy metabolism [53]. Consequently, increased autophagy [54] and decreased fibrosis [55] are thought to play an important role in the cardioprotective potential of AMPK activation. Specifically, AMPK phosphorylates the mammalian Unc-51 like autophagy activating kinase (ULK1) at Ser317 and Ser777, promoting autophagy, whereas mTOR, which is inhibited by AMPK, phosphorylates UKL1 on Ser757 and inhibits autophagy by disrupting the AMPK/ULK1 pathway [56]. TGFβ signalling increases the expression of SMAD2/3 transcription factors, leading to dedifferentiation of cardiac fibroblasts into cardiomyoblasts, which in turn secrete extracellular matrix proteins leading to cardiac fibrosis [57] and AMPK negatively regulates TGFβ signalling to act anti-fibrotically [58].

### The Role of AMPK in DOX Cardiotoxicity

The first evidence of the important role of AMPK inactivation in DOX cardiotoxicity comes from a study by Tokarska-Schlattner et al. in 2005 [59]. Short exposure (1 h) of isolated perfused rat hearts to low doses of DOX (2 μM), representative of plasma levels in DOX-treated patients (~ 600 ng/mL 45 min after infusion of 60 mg/m^2^, equivalent to ~ 1 μM [60]), led to reduced AMPK protein levels and AMPK phosphorylation as well as reduced phosphorylation of downstream targets such as acetyl-CoA carboxylase (ACC). This occurred before the onset of cardiac dysfunction and strongly suggests that perturbed cardiac energy homeostasis is involved in the development of DOX cardiotoxicity. In a separate study, DOX was also shown to inhibit AMPK protein expression and phosphorylation in the rat heart through DNA damage–induced Akt signalling, which activates mTOR signalling in a negative feedback loop and leads to cardiac remodelling [61]. While the aforementioned studies measured AMPK activation of the α subunit without isoform specificity, a different study in rats showed decreased mRNA expression of AMPKα2, the cardiomyocyte-specific isoform, upon chronic intravenous DOX treatment (weekly 3 mg/kg for 4 weeks) [62]. However, there is some discrepancy in the literature with respect to AMPK activation upon DOX treatment. For example, DOX was found to increase protein expression of AMPKα2 in mouse embryonic fibroblasts and mouse liver, leading to apoptosis via E2F1 signalling, whereas DOX decreased expression of AMPKα1 in the same models [63]. Furthermore, DOX was shown to induce AMPK activation (isoform unspecific) in rat H9c2 cardiomyoblasts [64]. Lastly, in a study of mice treated with a single high dose of intraperitoneal DOX (15 mg/kg), AMPK expression was increased and this was hypothesised to be due to decreased electron transport chain activity and reduced levels of ATP [65]. Since there are studies showing AMPK activation or inhibition of both the α1 and α2 isoforms of AMPK, isoform specificity cannot explain this discrepancy. However, one factor accounting for opposing findings across different studies may be the vast array of model systems (e.g. mice, rats, cardiomyocytes, mouse embryonic fibroblast) and treatment schemes (ip, iv, single dose, chronic weekly dose, low dose, high dose), which are employed to assess DOX cardiotoxicity, which may yield different results that do not necessarily reflect molecular events with clinically relevant doses and models. However, most studies agree with cardiac AMPK inhibition in response to DOX. The exact mechanism of AMPK inactivation by DOX remains elusive, though the stress-inducible sestrin proteins have been shown to play a role [66]. Sestrins are involved in oxidative stress defence and regulate AMPK signalling [67] and loss of sestrin function renders mice more susceptible to DOX cardiotoxicity [66].

## AMPK-Activating Agents and Their Role in DOX Cardioprotection

### Overview of AMPK-Activating Agents

AMPK-activating agents are highlighted in Fig. 1 and include the commonly prescribed anti-diabetic drug metformin and cholesterol-lowering statins, which can activate AMPK via phosphorylation of Thr172 in the α1 subunit [68, 69]. AMPK phosphorylation by metformin and statins may be independent of the cellular ATP to AMP ratio [70], though the exact mechanism is unclear. Resveratrol, a polyphenolic anti-oxidant, has been shown to activate AMPK by both changing the cellular energy state and by activating its upstream kinase, LKB1, through deacetylation by sirtuin 1 [71]. The tissue ATP to AMP ratio can also be reduced by the PPARγ agonists, namely the anti-diabetic thiazolidinediones, which activates AMPK allosterically through increased levels of AMP [72]. Small molecule AMP-mimetics, for example by 5-aminoimidazole-4-carboxamide riboside (AICAR), can activate AMPK allosterically [73]. AMPK can furthermore be activated by a range of specific synthetic AMPK activators [74]. Lastly, the ATP to AMP ratio can be decreased by exercise [75] and caloric restriction [76], indicating an energy-deficient state which activates AMPK. All the above agents/methods have been shown to increase levels of AMPK and/or AMPK phosphorylation, and have been used in models of DOX cardiotoxicity, demonstrating cardioprotective effects. However, these cardioprotective effects, while mediated via mechanisms known to involve AMPK (such as autophagy), have not always been shown experimentally to act directly via AMPK. In the following sections, we will introduce each of these agents separately and outline studies that have trialled them in the context of DOX cardiotoxicity, whether they conclusively showed involvement of AMPK signalling (summarised in Table 1) or not. We hope that this summary of evidence will shed some light on the potential role that AMPK-activating agents may play in cardioprotection for DOX cardiotoxicity.

**Table 1:** Preclinical studies showing AMPK-mediated cardioprotective effects of different compounds in models of DOX cardiotoxicity

Compound	Mechanism of AMPK activation	Downstream effect mediating cardioprotection	Model system	Reference
Metformin	Decreased ATP to AMP ratio? ATP to AMP ratio–independent AMPKα1 phosphorylation?	Increased PDGFR expression and increased cell viability	Rat H9c2 cardiomyoblasts	[77]
		Decreased oxidative stress, prevention of PPARα-cyclophilin D interaction	Rat H9c2 cardiomyoblasts	[78]
	Via adiponectin receptor?	Decreases apoptosis	HL-1 mouse cardiomyocytes	[79]
		Decreased apoptosis and fibrosis	C57BL/6 and APN-SE mice	[80]
Resveratrol	Decreased ATP to AMP ratio? ATP to AMP ratio–independent AMPKα1 phosphorylation?	Decreased apoptosis and fibrosis	Wistar rats	[81]
		Decreased fibrosis via reduced TGFβ	F344 rats	[82]
	Sirtuin 1 activation and deacetylation of LKB1 leading to AMPKα1 phosphorylation	Decreased apoptosis	Rat H9c2 cardiomyoblasts	[83]
	Decreased ATP to AMP ratio? ATP to AMP ratio–independent AMPKα1 phosphorylation?	Decreased apoptosis	Rat H9c2 cardiomyoblasts	[84]
		Decreased apoptosis and increased autophagy via ULK1/mTOR	Rat H9c2 cardiomyoblasts	[85]
Quercetin	Increased expression of AMPKα2	Increased expression of PPARα and PCG-1α	Sprague–Dawley rats	[86]
AICAR	AMP-mimetic (allosteric AMPK activation)	Decreased p53-mediated apoptosis	Mouse embryonic fibroblasts	[87]
2-Deoxyglucose	Decreased ATP to AMP ratio	Decreased apoptosis and increased autophagy	Rat neonatal cardiomyocytes	[88]
FGF21	Via sirtuin 1 activation and deacetylation of LKB1 leading to AMPKα1 phosphorylation	Decreased apoptosis, inflammation and oxidative stress	Rat H9c2 cardiomyoblasts, adult mouse cardiomyocytes and 129S1/SyImJ mice	[89]
Liraglutide	? (GLP-1 agonist)	Reduced inflammation and necrosis via Akt signalling	Wistar rats	[90]
Exenatide	? (GLP-1 agonist)	Increased autophagy	Rat H9c2 cardiomyoblasts	[91]
Melatonin	?	Reduced mitochondrial oxidative stress and apoptosis, increased PGC1α expression	Rat H9c2 cardiomyoblasts and C57BL/6 mice	[92]
Oleuropein	?	Reduced apoptosis, oxidative stress and normalised protein synthesis	Wistar rats	[93]
Aspalathin	?	Decreased apoptosis and increased autophagy	Rat H9c2 cardiomyoblasts	[94]

### Metformin

Metformin is a first-line drug for type 2 diabetic patients and the main mechanism of metformin’s anti-diabetic action is believed to be inhibition of complex I in the electron transport chain, which in turn inhibits hepatic gluconeogenesis [68]. This is linked to a risk of lactic acidosis [95] and therefore, metformin administration can be dangerous in some patients and is usually not recommended for diabetic patients with heart failure [96]. A more recent opinion, however, considers the risk of metformin in diabetic patients with heart failure as minimal and suggests that the cardioprotective benefit outweighs this risk [97]. Indeed, the metabolic and anti-fibrotic effects of metformin on the failing heart have been reviewed, encouraging large-scale clinical trials in diabetic patients with heart failure [98]. Moreover, metformin improves cardiac LVEF and survival in mice with ischaemia-induced heart failure [99]. Part of metformin’s anti-diabetic action is thought to be due to AMPK activation, which may be independent of changes in the ATP to AMP ratio due to complex I inhibition [70]. Metformin furthermore has dose-dependent effects [100] and its mechanism of action can be AMPK dependent or AMPK independent [101]. Metformin has been show to activate AMPK in adipose tissue [102], skeletal muscle [103] and hepatocytes, which decreases the activity of acetyl-CoA carboxylase, thereby reducing fatty acid synthesis and improving fatty acid oxidation [104]. In the heart, metformin promotes autophagy by both cytoplasmic AMPKα1 and nuclear AMPKα2 pathways [105], while it attenuates cardiac fibrosis through the TGFβ-SMAD3 pathway independent of AMPK [106].

In relation to DOX cardiotoxicity, low-dose metformin showed protective effects on rat H9c2 cardiomyoblasts treated with DOX, and this was due to AMPK activation and increased expression of the platelet-derived growth factor receptor (PDGFR), which increased cell viability [77]. In another study using rat H9c2 cardiomyoblasts, metformin was able to counteract hydrogen peroxide–induced mitochondrial DNA damage, and this was due to the prevention of PPARα-cyclophilin D interaction due to AMPK activation [78]. Metformin was also shown to normalise markers of autophagy (LC3B-II and p62) in rats treated with DOX, which resulted in improved cardiac function [107]. However, while cardiac levels of phosphorylated AMPK appeared to be increased in this model, this was not statistically significant. Metformin has also been shown to activate cardiac AMPK downstream of adiponectin signalling from the adipose tissue, which conferred protection from DOX in mouse HL-1 cardiomyocytes [79] and in mouse hearts in vivo [80]. This may explain how AMPK activation can be achieved in the heart despite unaltered cardiac energy status (ATP to AMP ratio). However, doses of metformin that showed AMPK activation in preclinical studies are usually 2–3 orders of magnitude higher than clinical doses [108], which potentially precludes optimum AMPK-mediated cardioprotection in man. One phase II trial using metformin in breast cancer patients receiving DOX has previously been started but was terminated due to insufficient accrual of patients.^1^ Future clinical trials are warranted to assess the potential cardioprotective effect of metformin in DOX-treated cancer patients, though careful consideration of dose and treatment scheme have to be made to ensure maximal cardioprotective potential through AMPK activation without severe adverse effects.

### Statins

Statins are commonly prescribed lipid-lowering agents and they have been shown to activate AMPK in mouse myocardium by increased phosphorylation of Thr172 of the α1 subunit [69]. This activation of AMPK is dependent on reactive nitrogen species [109] and/or Rac1 [110], at least in endothelial cells. Rac1 inhibition was shown in a mouse model of DOX cardiotoxicity treated with pitavastatin, leading to attenuated myocyte apoptosis and improved contractile function [111]. In a study with lovastatin in DOX-treated mice, inhibition of Rac1 signalling could furthermore achieve a reduction in cardiac fibrosis [112]. Moreover, lovastatin showed beneficial effects in a mouse model of chronic DOX cardiotoxicity [113]. However, while markers of cardiac damage (e.g. BNP) could be reduced with lovastatin in this setting, ejection fraction could not be rescued. In a rat model of DOX cardiotoxicity, rosuvastatin co-administration showed beneficial effects on cardiac LV function and myocardial injury 4 weeks after treatment cessation [114]. Rosuvastatin moreover reduced DOX-induced pro-inflammatory effects in rats [115]. Atorvastatin in turn ameliorated oxidative stress and DNA and cellular damage in a mouse model of DOX cardiotoxicity [116]. Lastly, fluvastatin pretreatment in an acute model of DOX cardiotoxicity exerted protective effects on cardiac oxidative stress, inflammation and apoptosis [117]. None of the above-mentioned studies, however, looked at the mechanism of statin cardioprotection with respect to AMPK activation directly. AMPK involvement in at least some of these studies is, however, likely given the fact that (a) statins do activate AMPK [69] and (b) some of the downstream effects such as fibrosis are linked to AMPK signalling [55].

From a clinical perspective, it has been shown in a retrospective study of female breast cancer patients treated with anthracycline-based chemotherapy that uninterrupted statin use throughout the follow-up period (2.55 ± 1.68 years) was associated with a significantly lower hazard ratio (0.3) for new-onset heart failure hospitalisation [118]. The use of statins as a cardioprotective prophylactic treatment in cancer patients on anthracycline-based chemotherapy may therefore be beneficial and further clinical trials are warranted.

### Resveratrol

The polyphenolic stilbene 3,5,40-trihydroxy-trans-stilbene (resveratrol) is produced by a multitude of plants and is a constituent of red wine [119]. Concentrations of resveratrol used in research are far greater than doses achievable by diet alone [120], which makes nutritional health claims of foods containing resveratrol in the cardioprotective setting questionable. However, resveratrol has been implicated as a cardioprotective drug for many cardiovascular diseases both in preclinical studies [121] and in man [122], and this is thought to be largely due to its anti-inflammatory and anti-oxidant action [123]. Of specific interest for the topic of this review, resveratrol has been shown to lead to activation of AMPK and to reduce cardiac fibrosis and hypertrophy in a mouse model of pressure overload by transverse aortic constriction [124]. Moreover, resveratrol has shown cardioprotective effects in animal models of DOX-induced cardiotoxicity by mitigating oxidative stress and apoptosis, modulating cardiomyocyte autophagy and ameliorating fibrosis, and studies supporting this have been reviewed recently [125].

Specifically, resveratrol has been shown to decrease fibrosis in DOX-treated rats [81]. In cells, this was due to upregulation of sirtuin 1, with concomitant decrease in TGFβ signalling in cardiac fibroblasts [82] and in H9c2 cardiomyoblasts [83]. Sirtuin 1 is upstream of the AMPK-phosphorylating kinase LKB1, initiating its activation by deacetylation [126], which explains its cardioprotection action in DOX cardiotoxicity [127]. Resveratrol also protects rat H9c2 cardiomyoblasts from doxorubicin-induced apoptosis by increasing AMPK phosphorylation and decreasing p53 expression [84]. In the same model system, resveratrol reduced apoptosis and increased autophagy downstream of AMPK activation, leading to ULK1 phosphorylation and decreasing phosphorylation of mTOR [85]. Another study, however, reported an inhibitory effect of resveratrol on autophagy, leading to protection against DOX cytotoxicity in neonatal rat ventricular cardiomyocytes, and this was independent of both AMPK and mTOR signalling and attributed to inhibition of p70S6 kinase [128]. Resveratrol furthermore was shown to elicit an anti-apoptotic and pro-autophagic effect by inhibiting the E2F1/AMPKα2 and E2F1/mTOR pathways [129], which is counterintuitive, though AMPKα2 has been shown previously to account for the cytotoxic effects of DOX [63]. Another non-resveratrol polyphenolic compound, quercetin, also conferred cardioprotection in rats, and this was associated with increased expression of AMPKα2, PPARα and PCG-1α [86]. No clinical trials have been performed to date using resveratrol in cancer patients on DOX-based chemotherapy, though data from preclinical studies above suggest that resveratrol may have cardioprotective effects in DOX-treated cancer patients.

### Thiazolidinediones

Thiazolidinediones are a class of anti-diabetic drugs which are ligands of the peroxisome proliferator–activated receptor-γ (PPAR γ) [130]. Thiazolidinediones have also been shown to activate AMPK by different mechanisms [72, 131–133]. The thiazolidinedione troglitazone can increase mitochondrial biogenesis in cancer cells [134]. Deus et al. assessed whether troglitazone could decrease DOX toxicity in rat H9c2 cardiomyoblasts by increasing mitochondrial number by the same mechanism; however, with the incubation times and concentrations employed in this study, no increase in mitochondrial content could be achieved and no protective effects were observed [135]. A meta-analysis of clinical trials in type 2 diabetic patients on either rosiglitazone or pioglitazone therapy revealed an increased risk of developing congestive heart failure due to fluid overload [136] making their clinical use to prevent heart failure in cancer patients receiving DOX-based chemotherapy unlikely.

### AICAR

The AMP-mimetic 5-aminoimidazole-4-carboxamide riboside (AICAR) is the prototype adenosine-regulating agent for reducing myocardial ischemic injury [137] and was shown almost 40 years ago to improve purine nucleotide synthesis in both cardiac and skeletal muscles [138]. AICAR furthermore activates AMPK [73] due to its structural equivalence to AMP [139], which increases mitochondrial biogenesis [140] and oxidative metabolism [141]. AICAR also increases autophagy downstream of AMPK by inhibiting mTOR signalling, thereby preventing apoptosis and improving cardiac function in a model of endotoxin-induced myocardial inflammation [142]. AICAR administration also leads to a reduction in cardiac fibrosis as it inhibits TGFβ signalling, thereby preventing adverse cardiac remodelling due to pressure overload [143]. AICAR furthermore reduces lipid synthesis and increases fatty acid oxidation in the liver in an AMPK-dependent manner [144], which could lead to reduced lipotoxicity of the heart. Publications employing AICAR in the DOX cardiotoxicity setting are, however, sparse. One study used AICAR in DOX-treated mouse embryonic fibroblasts and H9c2 rat cardiomyoblasts, which reversed SIRT1 dysfunction and p53 accumulation, leading to reduced cell death [87]. However, no studies have been published to date using AICAR in animal models of DOX cardiotoxicity in vivo. AICAR has a short half-life and has to be administered intravenously, which along with some side effects such as hypoglycaemia and bradycardia makes its clinical use challenging [145], though new synthetic AICAR derivatives with increased stability and AMPK activation potential are being synthesised [146]. Furthermore, AICAR has AMPK-independent effects, which means that off-target effects are likely, making specific AMPK activators potentially more attractive for clinical use [147].

### Exercise

Several preclinical studies have employed exercise as a cardioprotective strategy in DOX cardiotoxicity, and those studies are summarised in a recent review [148]. However, the cardioprotective mechanisms of exercise discussed in reference [148] are not directly attributed to AMPK activation. For example, cardioprotection in a mouse model of DOX cardiotoxicity with endurance treadmill exercise was correlated with increased autophagy and the authors conclude that this was despite no changes to AMPK/ULK1/mTOR signalling [149]. However, autophagy has been shown previously to be regulated by AMPK/ULK1/mTOR signalling [56]. Furthermore, the data in reference [149] does show a normalisation of ULK1 protein levels and mTOR phosphorylation with exercise, as well as an increase in phosphorylated AMPK, albeit the latter was not statistically significant. No other preclinical studies have directly assessed AMPK activation status in the heart to correlate with the cardioprotective findings of exercise. Clinical studies assessing the potential cardioprotective effects of exercise in heart failure in general as well as the cellular and molecular basis of such cardioprotective effects are summarised in reference [150]. In relation to DOX cardiotoxicity, an exercise bout performed 24 h prior to doxorubicin treatment reduced circulating NT-proBNP and increased systolic function in female breast cancer patients indicative of reduced acute DOX cardiotoxicity [151]. However, in the same patient group, exercise did not have an effect on markers of subclinical cardiotoxicity 7–14 days after DOX, even though there was a positive systemic effect on haemodynamics, musculoskeletal symptoms, mood and body weight [152]. The intensity and timing of exercise with respect to DOX treatment may alter what cardioprotective effect can be achieved and it may be beneficial to assess different exercise protocols in cancer patients on DOX chemotherapy.

### Dietary Restriction

The ATP to AMP ratio is indicative of the cellular energy state and can be modulated by dietary restriction [153]. Moderate diet restriction has also been shown to increase AMPK expression, fatty acid oxidation rates, ATP levels and cardiac function in rats treated with a single high dose of intraperitoneal DOX compared with rats on an ad libitum diet [154]. AMPK activation, reduced apoptosis and increased autophagy could furthermore be achieved in DOX-treated rat neonatal cardiomyocytes with the caloric restriction mimetic 2-deoxyglucose [88]. In addition, voluntary exercise (wheel running) in combination with caloric restriction reduced cardiotoxicity in DOX-treated rats, though AMPK expression and phosphorylation status or downstream effectors were not assessed in this study [155]. Contrary to the above, DOX has also been shown to supress the ULK1 pathway through inhibition of AMPK, leading to diminished autophagy, which could be reversed by prior starvation [156]. Given that cancer patients already suffer from their primary disease and the side effects of chemotherapy, it is questionable whether caloric restriction would be a sensible cardioprotective strategy for these patients.

### Specific AMPK Agonists

All AMPK-activating agents/strategies above also have off-target effects, and the exclusive role of AMPK activation for cardioprotection in DOX cardiotoxicity is challenging to ascribe. However, specific AMPK agonists have also been developed, which are targeted to AMPK directly and therefore show less off-target effects. One of these agents is the pan-AMPK activator, MK-8722 [157], which activates all 12 mammalian AMPK complexes. MK-8722 was shown to activate AMPK in rodent and rhesus monkey skeletal muscle, which led to insulin-independent glucose update in the skeletal muscle but was associated with cardiac hypertrophy [158]. The adenosine derivative and AMPK activator IMM-H007 furthermore showed decreased cardiac fibrosis and increased cardiac ejection fraction in a mouse model of angiotensin II-induced cardiac remodelling [159]. Another AMPK-activating agent is compound A-769662 [160], which activates AMPK both allosterically and by inhibiting dephosphorylation at the α subunit [161]. This compound shows AMPK activation of partially purified rat liver AMPK, inhibits fatty acid synthesis in rat hepatocytes and decreases liver malonyl-CoA levels in rats in vivo, indicating inhibition of ACC downstream of AMPK. A-769662 furthermore showed protection against ischaemia-reperfusion injury in a study in mice [162]. Interestingly, A-769662 on its own cannot increase glucose uptake in the heart, but shows synergistic effects with other AMPK activators, such as metformin, which potentiates the AMPK-activating effect and leads to protection from ischaemia-induced ROS formation and cell death [163]. A-769662 has not been tested in models of DOX cardiotoxicity, though its interaction with epirubicin on breast cancer cells has been assessed in comparison with metformin [164]. However, compound A-769662 has poor bioavailability and thus may not be useful in clinical settings, requiring long-term daily administration. Further β1-selective, γ-selective or pan-AMPK activators have been developed and tested in cancer models and the kidney but not the heart [74] or are in development and have not yet been tested in vivo [165]*.* It would be interesting to test some of these specific AMPK activators in models of DOX cardiotoxicity to more directly establish the role of AMPK activation in cardioprotection without any off-target effects.

### AMPK Activation by Other Compounds

A substantial list of compounds not classically associated with AMPK activation have been shown to have AMPK-mediated cardioprotective effects on DOX cardiotoxicity. For example, fibroblast growth factor 21 (FGF21), a regulator of glucose and lipid metabolism, could elicit cardioprotective effects in DOX-treated mice [89]. FGF21 led to AMPK activation by a cascade involving sirtuin 1 activation and deacetylation of LKB1, rendering it active to phosphorylate AMPK, which in turn was able to reduce inflammatory factors, oxidative stress and apoptosis. A promising new class of anti-diabetic drugs, the sodium-glucose cotransporter-2 (SGLT2) inhibitors, have shown improved cardiovascular outcomes in type 2 diabetic patients [166]. Empagliflozin is an SGLT2 inhibitor which dramatically reduced hospitalisation due to heart failure in diabetic patients [167]. Different SGLT2 inhibitors have moreover been shown to activate AMPK in cardiofibroblasts [168] and adipocytes [169]. Two recent studies furthermore showed that SGLT2 inhibitors preserve heart function in mice [170] and rats [171] treated with DOX. Another anti-diabetic drug, liraglutide, a glucagon-like peptide-1 (GLP1) analogue, protected rats from DOX-induced inflammation and necrosis by activating AMPK and the Akt signalling pathway [90]. Increased autophagy and reduced apoptosis could also be achieved with another GLP1 analogue, exanitide, in a rat model of DOX cardiotoxicity, where AMPK-mediated increase in autophagy improved cardiac LVEF [91]. The peptide hormone ghrelin showed cardioprotection by inhibiting autophagy through AMPK inhibition [172], which is counter to most studies attributing AMPK activation rather than inhibition to confer cardioprotection. In this study, AMPK phosphorylation was increased by DOX, though the treatment scheme both in their cell and in vivo models was far more severe than usually employed methods (10 μM DOX in cells and 4 weekly intraperitoneal injections of 8 mg/kg DOX in mice), which may explain the counterintuitive findings. Recently, the endogenous sleep-regulating hormone melatonin, which also has anti-oxidant capacity, was shown to reduce DOX cardiotoxicity in both rat H9c2 cardiomyoblasts and in C57BL/6 mice, via upregulation of AMPK/PGC1a signalling [92]. Oleuropein, which is a natural phenolic compound, reduced DOX-induced cardiotoxicity in Wistar rats and this was attributed to AMPK activation and a reversal of oxidative stress, apoptosis and impaired protein synthesis [93]. The rooibos flavonoid aspalathin also protects rat H9c2 cardiomyoblasts from DOX-toxicity by reducing apoptosis in an AMPK- and p53-dependent manner [94]. Other compounds based on traditional Chinese medicines such as *Astragalus membranaceus* [173], fermented *Cordyceps sinensis* [174], isodunnianol [175] and DT-010 [176] also showed AMPK-mediated cardioprotective effects in cell and mouse models of DOX cardiotoxicity. Other agents including salicylate [177], the endogenous and systemic anti-coagulant activated protein C [178] and the macrophage migration inhibitory factor [179], are also known to activate AMPK but have not been trialled in DOX cardiotoxicity.

## Conclusion

More than 50,000 women are diagnosed with breast cancer in the UK every year and ~ 30% of breast cancer patients are treated with anthracycline-based chemotherapy [180]. Breast cancer 10-year survival rates in the UK are now at 80% [2], and female breast cancer survivors are the most common patient group to suffer from DOX-HF. DOX cardiotoxicity is likely mediated by a combination of mechanisms, and some of these are linked by the cellular master regulator, AMPK. Cardioprotection through AMPK activation could, indeed, be achieved in preclinical studies with some AMPK-activating agents, such as metformin, statins and resveratrol as outlined in this review. None of these treatments though has reached clinical practise or even clinical trials until now, bar one phase II trial in breast cancer patients on DOX-based chemotherapy with metformin, though this trial was terminated due to insufficient patient accrual.^2^ The multitude of animal and cell models and varying concentrations and timescales of DOX used to study both the mechanism of DOX cardiotoxicity and the effect of AMPK-activating agents in this setting make it difficult to find consensus on molecular mechanisms. Furthermore, most of the AMPK-activating agents have off-target effects and are not AMPK specific. It may be useful to employ specific AMPK activators in comparison with commonly used agents with AMPK-activating capacity, such as metformin, statins and resveratrol, in models of DOX cardiotoxicity in order to definitively ascribe their cardioprotective roles to AMPK activation and to push them closer to clinical use. In addition to their cardioprotective function, statins have shown anti-tumour activity in three different mouse tumour models [181]. This may be, at least in part, due to AMPK activation, as different strategies to activate AMPK have been shown to provide anti-cancer activity in a range of cancer models [182, 183]. Strikingly, the AMPK-activating agents, metformin and AICAR have been shown to both attenuated cardiotoxicity and to reduce chemotherapy resistance [184] and proliferation [185] in human breast cancer cells and both AICAR and metformin can be safely administered alongside chemotherapy in patients [186, 187]. This makes their use as cardioprotective agents in cancer patients on DOX-based chemotherapy all the more attractive and feasible in the future.

## Footnotes

1 https://clinicaltrials.gov/ct2/show/NCT02472353?term=metformin&cond=Cardiotoxicity&rank=1

2 https://clinicaltrials.gov/ct2/show/NCT02472353?term=metformin&cond=Cardiotoxicity&rank=1.
